# Lack of Association of MiR-34b/c Polymorphism (rs4938723) with Hepatocellular Carcinoma: A Meta-Analysis

**DOI:** 10.1371/journal.pone.0068588

**Published:** 2013-07-31

**Authors:** Tie-Jun Liang, Hong-Jun Liu, Xiao-Qian Zhao, Cui-Hua Yu, Chen-Sheng Li

**Affiliations:** Department of Digestive Diseases, Provincial Hospital Affiliated to Shandong University, Jinan, Shandong Province, P.R. China; University of North Carolina School of Medicine, United States of America

## Abstract

**Background:**

Previous studies have focused on the association of miR-34 family members with carcinogenesis of many cancers, including hepatocellular carcinoma (HCC). It has been suggested that miR-34b/c polymorphism (rs4938723) is associated with susceptibility to HCC. In the present study, we performed a meta-analysis to systematically summarize the possible association between rs4938723 and the risk for HCC.

**Methodology/Principal Findings:**

We conducted a search of case-control studies on the associations of rs4938723 with susceptibility to HCC in PubMed, EMBASE, ISI Web of Science, Cochrane Central Register of Controlled Trials, ScienceDirect, Wiley Online Library, Wangfang database in China, and Chinese National Knowledge Infrastructure databases. Data from eligible studies were extracted for meta-analysis. HCC risk associated with rs4938723 was estimated by pooled odds ratios (ORs) and 95% confidence intervals (95% CIs). 3 studies on rs4938723 were included in our meta-analysis. Our results showed that neither allele frequency nor genotype distribution of the rs4938723 was associated with risk for HCC in all genetic models.

**Conclusions/Significance:**

This meta-analysis suggests that rs4938723 is not associated with the risk of HCC. Well-designed studies with larger sample size and more ethnic groups are required to further validate the results.

## Introduction

Hepatocellular carcinoma (HCC) is the most common primary malignant cancer of the liver and the third leading cause of death from cancer worldwide. Epidemiological survey suggests that East and South-East Asia and Middle and Western Africa have the highest prevalence of HCC with almost half of the new cases and deaths in China [1]. In HCC carcinogenesis, the molecular basis and genetic changes result in a specific phenotype often associated with varying tumor behaviors relevant to the prognosis and response to specific therapies [2]. MicroRNAs (miRNAs) are small, single-stranded, 19–21 nucleotide long non-protein-coding RNA molecules, functioning as negative regulators that involve post-transcriptional gene expression through binding to their target mRNAs regions and consequently lead to mRNA cleavage or translational repression [3]. Single nucleotide polymorphisms (SNPs) in miRNA-coding genes may have effects on either the expression or the function of miRNAs by altering the secondary structure of miRNA precursors, consequently leading to the aberrant expression of a series of target genes and contributing to cancer susceptibility [4]. Several studies indicated that miR-34b/c gene polymorphism was associated with HCC [5]–[7]. Since the relatively small sample size of a single study may not have enough power to detect slight effects of rs4938723 on HCC, meta-analysis may provide more credible evidence by systematically summarizing existed data. In this study, we have extensively reviewed literature and performed a meta-analysis based on all eligible case-control published data to evaluate the association between rs4938723 and cancer susceptibility.

## Methods

### Searching

We carried out a publication search in PubMed, EMBASE, ISI Web of Science, Cochrane Central Register of Controlled Trials, ScienceDirect, Wiley Online Library, Wangfang database in China, and Chinese National Knowledge Infrastructure (CNKI) databases with the following search terms: (miR-34b/c OR rs4938723) AND (hepatocellular carcinoma OR liver cancer OR hepatoma), by two independent investigators. Publication date and publication language were not restricted in our search. Reference lists were examined manually to further identify potentially relevant studies. All studies matching the inclusion criteria were retrieved for further examination and data extraction. All of the investigators have received training in literature search, statistics and evidence-based medicine.

### Selection

Studies included in this study must meet all the following criteria: (1) evaluated the associations between the rs4938723 and susceptibility to HCC, (2) studied on human beings, (3) in a case-control design, (4) detailed genotype data were provided for the calculation of odds ratio (OR) and 95% confidence interval (95% CI). We assessed the methodological qualities of included studies by the description of study population, detailed genotyping methods, the set of controls and cases and related statistical methods.

### Data extraction

For each study, the following characteristics were extracted: the first author's last name, year of publication, country of origin, ethnicity, the numbers of genotyped cases and controls, source of control groups, and genotyping methods.

### Statistical analysis

Meta-analysis was performed by using RevMan 5.0 software provided by the Cochrane Collaboration. We directly used Q-test and I^2^ test to examin the heterogeneity between each study. By heterogeneity test, if P>0.05, we select the Fixed Effect Mode1, and if P<0.05, we select the Random Effect Mode1 to merge HR. P<0.05 was considered as significant difference. Analysis of sensitivity includes the difference of point estimation and confidence intervals of the combined effects value at a different model, to observe whether it changes the result. To test the publication bias, we used the RevMan 5.0 statistical software to make the funnel plot. The rs4938723 was tested for the associations with HCC susceptibility based on different genetic models. The meta-analysis examined the overall association of the rs4938723 with the risk of HCC measured by odds ratios (ORs) at the 95% confidence intervals (CIs). To contrast the wild-type homozygote (TT), we first estimated the risk of the rare allele homozygote (CC) and heterozygous (TC) genotypes on HCC, then evaluated the risk of HCC under a dominant model (CC+TC vs. TT) and a recessive model (TT+TC vs. CC). The statistical significance of the pooled OR was determined with the Z test, and a P value of <0.05 was considered significant.

## Results

### Study Characteristics

**Figure 1:**
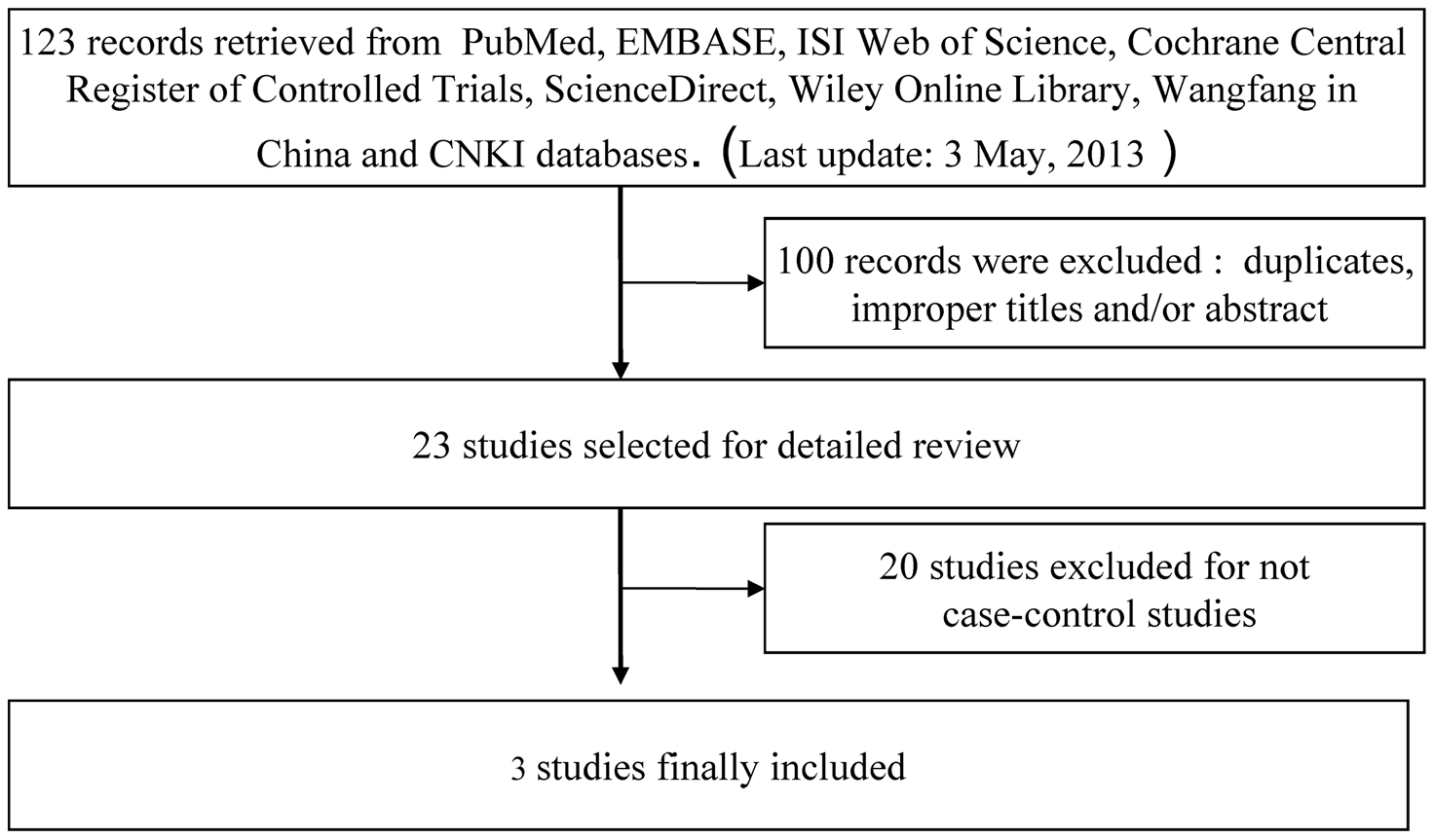
Flow diagram of study identification.

A total of 123 articles were retrieved after first search in PubMed, EMBASE, ISI Web of Science, Cochrane Central Register of Controlled Trials, ScienceDirect, Wiley Online Library, Wangfang in China and CNKI databases. As shown in Figure 1, after our selection, 3 case-control studies fulfilled the inclusion criteria [5]–[7]. The qualities of the studies were considered acceptable for our meta-analysis. Characteristics of included studies are summarized in Table 1. A total of 3 studies involving 1, 672 cases and 1,749 controls for rs4938723 were ultimately analyzed in our meta-analysis. There were 2 studies [5], [6] carried out in Chinese population while 1 study [7] was from South Korea population. Two genotyping methods were employed in the studies including polymerase chain reaction-restriction fragment length polymorphism (PCR-RFLP) and fluorescent-probe real-time quantitative PCR. The genotypes distribution in the controls was in agreement with HWE in all of the included studies.

**Table 1:** Characteristics of studies included in the meta-analysis.

Author	Year	country	Ethnicity	Genotyping methods	No. (Cases/controls)	Genotypes Case (%)			Genotypes Contol (%)		
						TT	TC	CC	TT	TC	CC
XU	2010	China	Asian	PCR-RFLP	502/549	204 (40.64)	236(47.01)	62(12.35)	266(48.45)	229(41.71)	54(9.84)
SON	2013	South Korea	Asian	PCR-RFLP	157/201	69(43.9)	75(47.8)	13(8.3)	110(54.7)	74(36.8)	17(8.5)
HAN	2013	China	Asian	Fluorescent-probe real-time quantitative PCR	1013/999	451(44.52)	444(43.83)	118(11.65)	456(45.65)	424(42.44)	119(11.91)

### Meta-analysis

**Figure 2:**
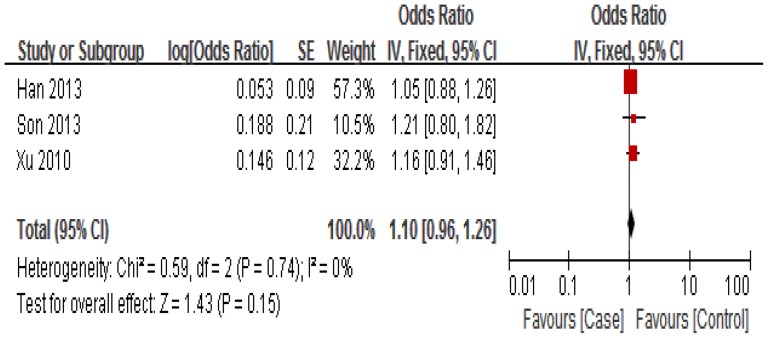
Forest plot of HCC risk associated with rs4938723 (dominant model: TC+CC vs TT). The squares and horizontal lines correspond to the study-specific OR and 95% CI, respectively. The area of the squares reflects the study-specific weight. The diamond represents the pooled results of OR and 95% CI. In this analysis, fixed-effects model was used.

**Figure 3:**
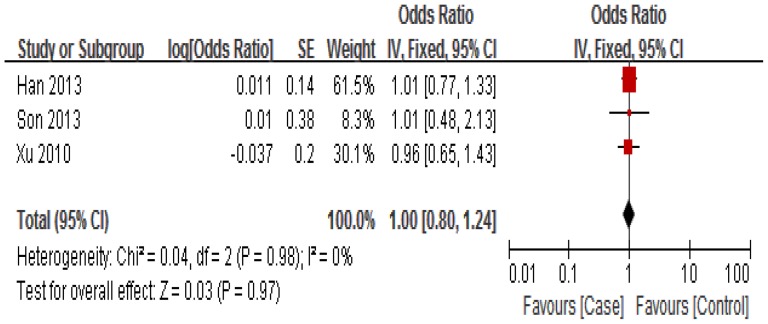
Forest plot of HCC risk associated with rs4938723 (Recessive model: TC+TT vs CC). The squares and horizontal lines correspond to the study-specific OR and 95% CI, respectively. The area of the squares reflects the study-specific weight. The diamond represents the pooled results of OR and 95% CI. In this analysis, fixed-effects model was used.

**Figure 4:**
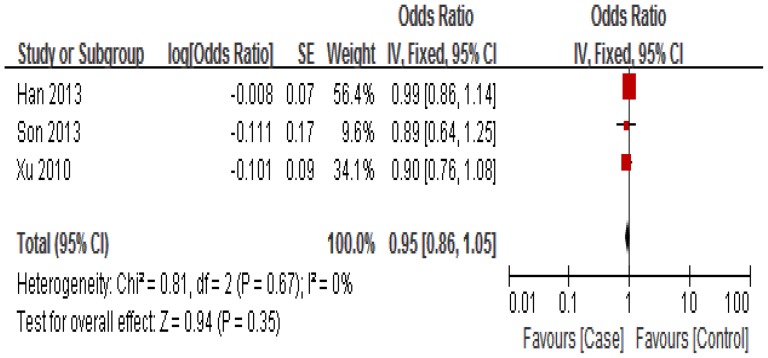
Forest plot of HCC risk associated with rs4938723 (Allele: T vs C). The squares and horizontal lines correspond to the study-specific OR and 95% CI, respectively. The area of the squares reflects the study-specific weight. The diamond represents the pooled results of OR and 95% CI. In this analysis, fixed-effects model was used.

The association between miR-34b/c rs4938723 polymorphism and susceptibility to HCC was analyzed in four independent studies with 1, 672 cases and 1,749 controls. Results of the meta-analysis are shown in Figure 2 Figure 3 and Figure 4. Q-test in all of the genetic models showed no significant heterogeneity. Therefore, fixed-effects model was used to analysis the association. No significant association between miR-34b/c rs4938723 polymorphism and susceptibility to HCC was identified in any of the genetic models (T versus C: OR = 0.95, 95% CI 0.86–1.05, P = 0.35; [TC+CC] versus TT: OR = 1.10, 95% CI 0.96–1.26, P = 0.15; CC versus [TC+TT]: OR = 1.00, 95% CI 0.80–1.24, P = 0.97).

### Publication Bias

**Figure 5:**
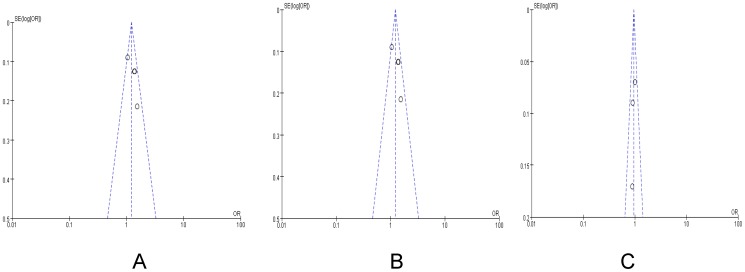
Begg's funnel plot for publication bias test. Each circle denotes an independent study for the indicated association. Log[OR], natural logarithm of OR. Horizontal line stands for mean effect size. A: dominant model, B: Recessive model, C: Allele.

Funnel plot and Egger's test were performed to assess the publication bias of the literature. Symmetrical funnel plots were obtained in the SNP tested in all of the models. Egger's test further confirmed the absence of publication bias in this meta-analysis (P>0.05) (Fig. 5).

### Sensitivity Analysis

We deleted one single study from the overall pooled analysis each time to check the influence of the removed data set to the overall ORs. The pooled ORs and 95% CIs were not significantly altered when any part of the study was omitted, which indicated that any single study had little impact on the overall ORs.

## Discussion

In the present meta-analysis, we did not find that rs4938723 in the promoter region of pri-miR-34b/c was associated with an increased risk of HCC. The SNP rs4938723 (T>C) located within the CpG island of pri-miR-34b/c and might create a predicted GATA-binding site, and therefore, it may affect miR-34b/c expression by both genetic and epigenetic mechanisms. Ectopic miR-34b/c caused cell cycle arrest in the G1 phase [8] and miR-34b/c inhibited proliferation and colony formation in soft agar [9]. In a previous study, down-regulation of mir-34b/c by methylation was found in colorectal cancer [10], oral cancer [11], and malignant melanoma [12]. And serveral previous studiese reported the rs4938723 in the promoter region of pri-miR-34b/c associated with HCC in the Chinese [5], [6] and the Korean [7].

In this meta-analysis, a total of 3 case-control studies were analyzed to provide a comprehensive assessment of the association between miR-34b/c rs4938723 polymorphism and HCC. Genotypes in all studies were detected with genetic DNA from blood samples using 2 genotyping methods totally. All of the studies checked genotypes for quality control. Genotype distribution of controls in all studies was consistent with HWE. By the meta-analysis, our results did not support a genetic association between rs4938723 and susceptibility to HCC.

Exploring heterogeneity is one of the important goals of meta-analysis [13]. In the present study no significant heterogeneity was found among the included studies (P = 0.62, I^2^ = 0%). Sensitivity analysis also showed that omission of any single study did not have significant impact on the combined ORs. Furthermore, funnel plot did not reflect obvious asymmetry, and Egger's test further indicated no considerable publication bias in this meta-analysis. This made the results of this meta-study more reliable to some extent.

Be that as it may, there remained some limitations in this meta-analysis. In the studies included, the genotyping methods used were not the same. Besides, other clinical factors such as age, sex and different chemotherapies in each study might lead to bias. Determining whether or not these factors influence the results of this meta-analysis would need further investigation.

In conclusion, our study suggested that SNP rs4938723 in the promoter region of pri-miR-34b/c was associated with a significantly increased risk of HCC. Larger well-designed epidemiological studies with ethnically diverse populations and functional evaluations are warranted to confirm our findings.

## References

[1] El-Serag HB, Rudolph KL (2007) Hepatocellular carcinoma: epidemiology and molecular carcinogenesis. Gastroenterology 132: 2557–2576. 17570226 10.1053/j.gastro.2007.04.061

[2] El-Serag HB (2011) Hepatocellular carcinoma. N Engl J Med 365: 1118–1127. 21992124 10.1056/NEJMra1001683

[3] Bartel DP (2004) MicroRNAs: genomics, biogenesis, mechanism, and function. Cell 116: 281–297. 14744438 10.1016/s0092-8674(04)00045-5

[4] Saunders MA, Liang H, Li WH (2007) Human polymorphism at microRNAs and microRNA target sites. Proc Natl Acad Sci U S A 104: 3300–3305. 17360642 10.1073/pnas.0611347104 PMC1805605

[5] Son MS, Jang MJ, Jeon YJ, Kim WH, Kwon CI, et al (2013) Promoter polymorphisms of pri-miR-34b/c are associated with hepatocellular carcinoma. Gene doi:10.1016/j.gene.2013.04.042 10.1016/j.gene.2013.04.042 23632240

[6] Han Y, Pu R, Han X, Zhao J, Zhang Y, et al (2013) Associations of pri-miR-34b/c and pre-miR-196a2 polymorphisms and their multiplicative interactions with hepatitis B virus mutations with hepatocellular carcinoma risk. PLoS One 8 3: e58564 doi:10.1371/journal.pone.0058564 23516510 10.1371/journal.pone.0058564 PMC3596299

[7] Xu Y, Liu L, Liu J, Zhang Y, Zhu J, et al (2011) A potentially functional polymorphism in the promoter region of miR-34b/c is associated with an increased risk for primary hepatocellular carcinoma. Int J Cancer 128 2: 412–7 doi:10.1002/ijc.25342 20309940 10.1002/ijc.25342

[8] Xiang Y, Fan S, Cao J, Huang S, Zhang LP (2012) Association of the microRNA-499 variants with susceptibility to hepatocellular carcinoma in a Chinese population. Mol Biol Rep 39: 7019–7023. 22311030 10.1007/s11033-012-1532-0

[9] Akkiz H, Bayram S, Bekar A, Akgollu E, Ulger Y (2011) A functional polymorphism in pre-microRNA-196a-2 contributes to the susceptibility of hepatocellular carcinoma in a Turkish population: a case–control study. J Viral Hepat 18: e399–e407. 21692953 10.1111/j.1365-2893.2010.01414.x

[10] Toyota M, Suzuki H, Sasaki Y, Maruyama R, Imai K, et al (2008) Epigenetic silencing of microRNA-34b/c and B-cell translocation gene 4 is associated with CpG island methylation in colorectal cancer. Cancer Res 68: 4123–4132. 18519671 10.1158/0008-5472.CAN-08-0325

[11] Kozaki K, Imoto I, Mogi S, Omura K, Inazawa J, et al (2008) Exploration of tumor suppressive microRNAs silenced by DNA hypermethylation in oral cancer. Cancer Res 68: 2094–2105. 18381414 10.1158/0008-5472.CAN-07-5194

[12] Lujambio A, Ropero S, Ballestar E, Fraga MF, Cerrato C, et al (2007) Genetic unmasking of an epigenetically silenced microRNA in human cancer cells. Cancer Res 67: 1424–1429. 17308079 10.1158/0008-5472.CAN-06-4218

[13] Petitti DB (2001) Approaches to heterogeneity in meta-analysis. Stat Med 20: 3625–3633. 11746342 10.1002/sim.1091

